# A comparative study: do “clickers” increase student engagement in multidisciplinary clinical microbiology teaching?

**DOI:** 10.1186/s12909-017-0906-3

**Published:** 2017-04-08

**Authors:** Niall T. Stevens, Hélène McDermott, Fiona Boland, Teresa Pawlikowska, Hilary Humphreys

**Affiliations:** 1 Department of Clinical Microbiology, Royal College of Surgeons in Ireland, RCSI Education and Research Centre, Beaumont Hospital, Beaumont, Dublin 9, Ireland; 2 Division of Population Health Sciences, Royal College of Surgeons in Ireland, Beaux Lane House, Lower Mercer Street, Dublin 2, Ireland; 3 RCSI Health Professions Education Centre, Royal College of Surgeons in Ireland, St. Stephen’s Green, Dublin 2, Ireland; 4 Department of Microbiology, Beaumont Hospital, Beaumont, Dublin 9, Ireland

**Keywords:** Clinical microbiology, Multidisciplinary, “Clickers”, Medical education

## Abstract

**Background:**

Audience response devices, or “clickers”, have been used in the education of future healthcare professionals for several years with varying success. They have been reported to improve the learning experience by promoting engagement and knowledge retention. In 2014, our department evaluated the use of “clickers” in a newly introduced multidisciplinary approach to teaching large groups of third year medical students clinical cases developed around a microbiology theme.

**Methods:**

Six multidisciplinary teaching sessions covering community-acquired pneumonia, tuberculosis, infective endocarditis, peritonitis, bloodstream infection with pyelonephritis and bacterial meningitis were included in the study. Three involved the use of the “clickers” and three did not. Consenting undergraduate students attended the designated classes and afterwards answered a short online quiz relating to the session. Students also answered a short questionnaire about the “clickers” to gauge their attitudes on the use of these devices.

**Results:**

Of 310 students, 294 (94.8%) agreed to participate in the study. Interestingly, the grades of online quizzes after a session where a “clicker” was used were slightly lower. Looking only at the grades of students who engaged completely with the process (*n* = 19), there was no statistical difference to suggest that the devices had a positive or negative impact on knowledge retention. However, student attitudes to using the devices were positive overall. Fifty-five percent strongly agreed and 27% agreed that teaching sessions where the “clickers” were used were more engaging. Thirty-four percent strongly agreed and 36% agreed that the “clickers” made important concepts more memorable and 54% felt the device enhanced their understanding of the topic being covered.

**Conclusions:**

Overall, it appears that “clickers” help in improving student engagement in large classroom environments, enhance the learning experience, and are received positively by medical students but their impact on knowledge retention is variable.

## Background

The traditional, didactic lecture is a common learning activity in medical education as it is an efficient, and economical, mechanism to transfer knowledge and fundamental concepts in medicine to large groups of students. However, lectures are not without their draw-backs. Students can find these very one directional, teacher-centric, passive and even monotonous [1, 2]. Despite the best efforts of the teacher to encourage students to focus and understand the core concepts during lectures, the actual format of the learning activity is thought to encourage a focus on the superficial learning points [3]. Furthermore, the lecture may not always suit the learning needs of all students and they may opt to not attend as a result. [4].

Encouraging active learning and making lectures, and other large group teaching sessions, engaging is now of great interest to health professions educators [5–8]. One widely used method involves “clickers”, which are small hand-held devices that students can use to respond to questions, most often multiple choice questions (MCQs), posed during a teaching session. These devices are increasingly being used in both large and small classes in many third level institutions to improve the learning experience [6, 8–11]. Generally they are used to introduce variety into a teaching session and to assess understanding of a topic in real-time [9]. The student’s response is received using wireless technology by the software in which the PowerPoint presentation was created and the combined responses from the whole class are compiled to create a bar chart. At this point the individual(s) delivering the session discuss the bar chart and explain why the answer options are correct or incorrect. Their application in the clinical teaching environment is also becoming more common and numerous studies have shown their use to be beneficial due to their ability to increase student engagement and to promote knowledge retention [12–15].

Multidisciplinary, and interprofessional, approaches to teaching students are also becoming more common and desirable within the health sciences as they seek to model real world interactions [16, 17]. One early study noted the benefits of a multidisciplinary approach in the delivery of paediatric pathology during a residency rotation and the authors suggested that this novel method of teaching could be applied to medical education as a whole to create a more informative and engaging experience [17]. More recently, an inter-disciplinary approach to the training of first year residents on a labour ward and delivery unit was evaluated by the Faculty of Midwives, the Department of Obstetrics and Gynaecology at the University of Colorado who found this was well received [16].

The Royal College of Surgeons in Ireland (RCSI) is a university level institution with over 2000 registered medical, pharmacy and physiotherapy students from over 60 countries. The Department of Clinical Microbiology delivers content across all three disciplines both online and through traditional modalities of didactic teaching, such as lectures and tutorials. In 2012, the Department led the introduction of a multidisciplinary teaching (MDT) session on peritonitis with the third year (Intermediate Cycle, IC) medical students. Since then five additional MDTs with a clinical microbiology theme have been introduced and they cover topics such as community-acquired pneumonia (CAP), infective endocarditis (IE), pulmonary tuberculosis (TB), viral hepatitis, bloodstream infection (BSI) with pyelonephritis, and bacterial meningitis. Depending on the case, and subject material, other teaching staff may include those from medicine, surgery, pathology and radiology/imaging. The aim of the MDT is to demonstrate to the students that the management of patients with complex or systemic infections requires a multi-disciplinary approach. The sessions highlight the key contributions of each discipline, at what stage this contribution is made and they demonstrate clearly the level of communication required between the disciplines when a management plan is being devised for the patient in the case presented. Case-based teaching is now a well established pedagogical tool in the health sciences. Students and teachers alike enjoy when the class centres around a case as it reflects the “real”-life environment and can enhance the learning experience but it should be noted that there is incomplete evidence to suggest that case-based teaching is better than any other method [18].

Student feedback from the first MDT session was very positive. However, a major issue with this method of large-group teaching was the lack of engagement between those delivering the session and the students. Furthermore, those delivering the teaching sessions felt that students did not wish to volunteer answers when questioned directly or they were reluctant to engage in discussion in the presence of so many of their peers. One study also reported that “clickers” provide a sense of anonymity that the students seem to prefer [19]. For these reasons, the Department of Clinical Microbiology decided to evaluate the use of “clickers” during these microbiology themed MDTs. Our overall aims were to assess the impact of the “clickers” on learning during our MDT sessions and to assess student attitudes to the use of these devices in their teaching.

## Methods

### Ethical approval & student recruitment

Ethical approval was sought from the RCSI Research Ethics Committee to collect data from the IC medical students in January 2014. The study took place over both semesters in this cycle and ended in December 2014. All consenting students were asked to complete quizzes associated with the study on the virtual learning environment, Moodle. Students were recruited before the first MDT when a short presentation and demonstration of the “clickers” was also given.

### Automated student response system

PowerPoint presentations addressing the intended learning outcomes with embedded interactive questions were prepared using the software obtained from Turning Technologies (Northern Ireland). The software allows for the creation of PowerPoint presentations with embedded questions such as multiple choice or true or false that can be posed to the students during class and polled in real-time. The students then use handheld “clickers” to assess their understanding by choosing the corresponding option on the key-pad of the device. The software allows the user to limit the length of time the polling of each question is open and the students can also see a count-down timer on the slide. When polling is closed, the software collates all responses to generate a graph indicating the percentage responses for each option. The software allows the user to indicate the correct answer using a variety of markers.

### Study design

This was a comparative observational study in which three MDTs (CAP, peritonitis and meningitis), spread over both semesters, involved the use of the “clickers” and three MDTs (TB, IE and BSI with pyelonephritis), spread over both semesters, took place without the use of the clickers. The MDT is a case-based large group teaching session that takes place in a lecture theatre and lasts approximately 90 min. MDTs with an infection theme are coordinated by the Department of Clinical Microbiology and the presentation and sessions are prepared by senior clinician academics. The MDT is case-based and problem-orientated. The students are presented with the patient’s history initially and the different disciplines take them through the various aspects of the case, where appropriate. For example, medicine will work through the differential diagnosis, then clinical microbiology will discuss specimen collection and the possible laboratory results, then possibly radiology would discuss the findings from imaging and then the case may revert to clinical microbiology and a discussion around appropriate antibiotics. Material covered in lectures is put into a clinical context at the MDT.

In each MDT, whether “clickers” were used or were not used, five MCQs that covered the same topics i.e. signs & symptoms, appropriate diagnostic tests, the most common causative pathogen, the most appropriate antimicrobial to treat the infection and the most appropriate prevention strategy/other appropriate management relating to the case, were posed at various stages throughout the MDT. When “clickers” were used the students answered the MCQ in real-time. The MCQ was then discussed to ensure that the reasons for the most appropriate correct answer were understood. When no “clickers” were used the MCQ was simply posed verbally to the class by the teacher and answered by a show of hands only. The most appropriate correct answer and wrong answers were again discussed for consistency in the non “clicker” sessions.

### To assess the impact of “clickers” on the learning

The “clickers” were not assigned to any one student but instead were collected prior to the commencement of class and during the taking of attendance. After each MDT, students were asked to answer the same five MCQs, as those posed in class, again *via* the virtual learning environment (online). Each student who had consented to participate was asked to complete the quiz over a 24 h period. A recent prospective cross-over interventional study assessing the impact of interactive lectures of biochemistry in a medical curriculum found that there was a statistically significant increase of comprehension in students who attended an interactive session compared to a non interactive session and that this was more evident when the topic covered was clinically orientated and when the students were assessed immediately after the session [7]. Our rationale was similar, although we did not assess immediately after, we did assess our student’s comprehension and simple recall within a 24 h period after the MDT, which was also clinically focused. For maximum effect on learning, students again were given instant feedback once they had completed and submitted the quiz online. Each quiz consisted of five MCQs all worth two marks. The highest possible grade was therefore ten. Examples of the MCQs posed in the class and online can be seen in Appendix.

### To assess student attitudes to the use of the “clickers”

On the same day of the last MDT, and immediately after the class had finished, a survey was conducted using the actual “clickers”. To comply with our ethical approval, to ensure student anonymity, for efficiency and to ensure maximum responses by avoiding survey fatigue, it was decided to conduct the student attitudes survey in this manner. Moreover, the Turning Technologies automated response system and software is designed for such purposes and to obtain immediate feedback. Students were asked to give their opinions on the use of the devices and the potential positive and negative impact they had on the learning environment. A 5-point Likert scale (strongly disagree to strongly agree) was used to assess student attitudes. The statements included in the study were as follows; (1) The “clickers” were easy to use; (2) MDTs were more enjoyable & interesting than normal lectures; (3) MDTs where “clickers” were used were more engaging than MDTs without “clickers”; (4) The use of a “clicker” during teaching distracted me from learning; (5) The use of a “clicker” enhanced my understanding of the topic being covered; (6) The use of the “clicker” during the MDT made important concepts more memorable.

Attendance was taken at all MDTs, including the last session where the survey was conducted. This facilitated the extraction of the demographic details of the cohort of students that participated in the survey.

### Statistical analysis

The responses to each question on the survey were summarised using percentages and bar charts.

For all six MDT sessions, the average online post-MDT grade for each student was calculated. Only students who completed at least one post-MDT quiz in which a device was used and one post-MDT quiz in which no devices were used were included in this analysis. To assess the impact of the “clickers” on knowledge retention, a paired t-test was used to compare the difference in the means of the post-MDT grades, in which devices were used, with those in which no devices were used. Furthermore, a paired t-test was conducted including only students who completely engaged in the process and completed all online post-MDT quizzes (i.e. completed all three post-MDT quizzes in which no devices were used and all three post-MDT quizzes in which devices had been used). All analysis was conducted using Strata version 14 [20].

## Results

### Student recruitment and engagement with study

**Fig. 1:**
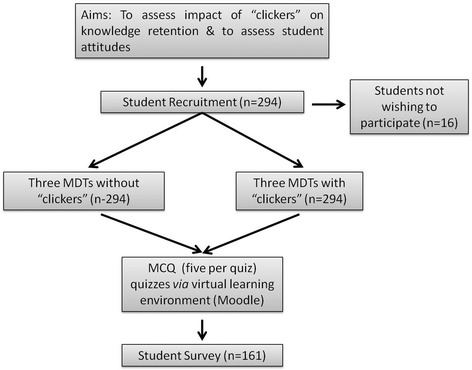
Student recruitment process and study design. Two-hundred and ninety four students consented to participate in the study. There were six MDTs in total in which three involved the use of the “clickers” and three did not. After each MDT, students were asked to answer the same five MCQs, as those presented in class, again online *via* the virtual learning environment. Each MCQ was worth two marks so the highest possible grade achievable was ten marks. Statistical analysis on student grades from the online quizzes was performed to assess the impact of the “clickers” on knowledge retention. Students then completed a survey to determine attitudes to the teaching environment and use of the devices. Student demographics of those in attendance (*n* = 161) on the day of the survey were also collected from student records

A total of 294/310 (94.8%) students consented to participate in the study (Fig. 1). The remaining 16 students did not wish to consent as they did not wish to have an extra workload or they simply did not engage in the process. One hundred and sixty one (55%) students who consented to participate in the study also participated in the student survey. These students were in attendance on the day the survey took place (Fig. 1).

The number of students who completed the online quizzes and the overall mean grades from each are summarised in Table 1. Initially, participation in the study was good with high numbers of students engaging with the online quizzes after the MDT with greater than 50% of those in attendance completing the online quiz. While attendance remained high at the MDTs over the duration of the study participation in the online quizzes fell below 40%, particularly near the end of semester two. Two of the MDTs where “clickers” were used took place in semester two, with the third MDT (bacterial meningitis) in which “clickers” were used taking place on the last day of the term and two weeks before the examinations. The low participation in the online quiz (49 (30.4%)) after the bacterial meningitis MDT session probably reflects this.

**Table 1:** Number of students participating and the mean marks of online quizzes

MDT	No. of students completing online quiz/ No. Students in attendance at MDT (%)	Mean Mark (±SD^a^)
*Semester 1*		
Community-acquired pneumonia^b^	268/294 (91.2)	8.13 (2.04)
Pulmonary tuberculosis	206/285 (72.3)	8.68 (1.57)
Infective endocarditis	149/274 (54.4)	8.30 (2.04)
*Semester 2*		
Peritonitis^b^	81/236 (34.3)	7.55 (2.02)
Bloodstream infection & pyelonephritis	81/284 (28.5)	6.91 (2.03)
Bacterial meningitis^b^	49/161 (30.4)	5.59 (1.58)

^a^Standard deviation

^b^Denotes MDT in which “clickers” were used

### Impact of “clickers” on student learning

A total of 225 participants completed at least one online quiz in relation to an MDT in which “clickers” had been used and one online quiz in relation to an MDT in which no “clickers” were used. The total number of quizzes completed by the 225 students is summarised in Table 2.

**Table 2:** Number of two or more online quizzes completed by students

Total number of online quizzes completed	Number of students
2	65
3	70
4	39
5	32
6	19

For each student, for the MDTs in which no “clickers” were used, and similarly for the MDTs in which “clickers” were used, the average online post-MDT grade was calculated. The overall mean grades of the online quizzes in relation to MDTs in which “clickers” were and were not used was 7.72 (SD:1.93) and 8.22 (SD:1.52), respectively. The difference in means between grades of quizzes in relation to MDTs in which “clickers” and no “clickers” were used was calculated and a t-test was used to compare the differences. Interestingly, there was evidence of a negative impact of “clickers” (*P* = 0.02) with a mean difference in scores of −0.5 (95% confidence interval: −0.80 to −0.19), indicating that on average students scored half a mark lower in the quizzes after MDTs in which “clickers” were used. However, when only students who completely engaged in the process and completed all online post-MDT quizzes are considered (*n* = 19), there was no evidence of a difference in the grades they obtained (*P* = 0.07).

### Student attitudes to “clickers” & MDTs

The mean age of the group was 22 (range 19 to 32) and most were female (Table 3). A large proportion of the respondents came from Australasia, Malaysia in particular, followed by the Middle East. In total, 115 (71%) students do not regard English as their first language.

**Table 3:** Demographics of 161 students participating in the survey

Demographic	No. of Students (%)/Age
*Sex*	
Male	68 (42.2)
Female	93 (57.8)
*Age*	
Mean (Range)	22 (19 to 32) years
*Region of Birth*	
Ireland & rest of Europe	21
North America & Caribbean	22
Middle East & Africa	30
Australasia	88

**Fig. 2:**
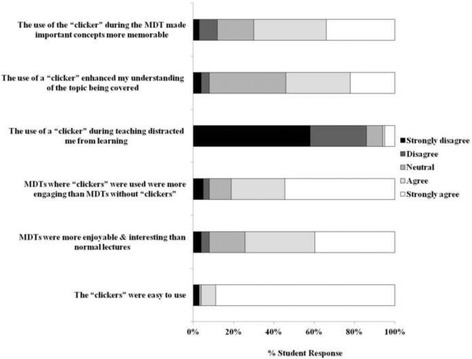
Student attitudes to “clickers” and MDTs. One hundred and sixty one students participated in the real time survey using the “clickers”. Students were asked their opinions in relation to the “clickers” and the MDT sessions. A 5-point Likert scale of strongly disagree to strongly agree was used to gauge student opinions. Data represents percentage number of students with a specific opinion relating to the statement posed

The majority of students (88%) found the devices easy to use and 75% strongly agreed or agreed that the MDT as a mode of teaching was more enjoyable and interesting than a normal didactic lecture (Fig. 2). The majority of students agreed (27%) or strongly agreed (55%) that MDTs where “clickers” were used were more engaging than MDTs where no “clickers” were used. Only 5% of the respondents strongly disagreed with their classmates (Fig. 2). Importantly, only 6% considered the “clickers” a distraction and 70% agreed or strongly agreed that the devices made important concepts more memorable. Of note, 54% agreed or strongly agreed that the devices were of some benefit to their educational experience by enhancing their understanding of the topic covered (Fig. 2).

## Discussion

As a mode of teaching for undergraduate medical students, the MDT helps show the professional interactions and multidisciplinary approach required in the management of patients with common infections. The majority of third year medical students polled in this study found the MDTs to be more enjoyable and interesting than their routine didactic lectures delivered by a single medical expert of one specific discipline. Several issues have been raised about the effectiveness of the lecture as a teaching activity. It is often said that the lecture is more teacher centred, that there are few opportunities for student reflection and that they do not promote problem-solving, patient management or the development of professional identity [21, 22]. It has also been said that lectures lacking interaction are not engaging enough for students to foster their critical skills [23] but do highlight the need for students to incorporate skills learned into their daily practices [24].

During the initial implementation of the MDT to our teaching program, when “clickers” were not used, we quickly identified limitations in a diverse and highly competitive student body. For example, it is known that aspiring surgeons are highly competitive [25]. Often, medical students do not wish to answer questions in front of their peers in case they are wrong. It is not surprising that the students (75%) in this study found the MDTs where “clickers” were used to be more enjoyable. These sessions most likely provided them with a “safe” learning environment and a sense of anonymity that students prefer [19]. Students also found the interactive “clicker” sessions to be more engaging than those when none were used. One recent study has shown that medical students studying biochemistry preferred interactive large group teaching sessions, such as lectures, to non interactive session [7]. This same study found that their interactive lectures enhanced understanding, created an interest in the lecture, motivated students to study, enhanced recollection and removed any doubts or misunderstandings [7]. However, this study did not describe the intervention that created the interactive learning environment. Importantly, 54% of the students surveyed in our study found the use of “clickers” and the additional interaction they created between the teacher and the class when the question and answers were discussed in detail enhanced their understanding. One study also found that students in a physician assistant program were more attentive when the devices were used but they also noted that the students found the teaching to be more enjoyable and engaging [26]. Another study also found that “clickers” made a lecture delivered to a variety of qualified healthcare professionals, which included clinicians, pharmacists and nurses, more interesting while still keeping their attention [14]. Furthermore, other studies have shown that “clickers” can promote advanced reasoning skills [27] and improve knowledge gain directly after teaching sessions [28]. In contrast to this, we saw no difference on retention of knowledge immediately after MDT between “clicker” and no “clicker” sessions. Similar to this, Duggan et al., [6] also saw no difference in MCQ scores from questionnaires based on lectures using “clickers” and normal lectures without the devices.

Most students who completed the online quizzes obtained between 80 and 100% (data not shown) grades on the same day after sessions regardless of whether a device was used or was not. This suggests their ability to, for example, make a differential diagnosis, or to identify the most likely causes of the infection or to develop a management plan, regardless of the topic being covered, was already well developed and understood. In fact, analysis of the data showed that there was evidence of a small negative impact on the grades of the whole class when the “clickers” were used. However, no positive or negative impact on the grades of the nineteen students who engaged with the entire study, and completed all six online quizzes, could be seen following statistical analysis. In a systematic review of 21 articles that evaluated the use of “clickers” in teaching, it was found that only fourteen identified a statistically significant positive impact on knowledge when the devices were used. [29] This would suggest our findings are not unusual in the context of large group teaching.

The lack of engagement with the online quizzes, particularly closer to the end-of-semester examinations and a lack of interest for non summative examinations hindered this study. Other studies have shown there to be no impact of the use of audience response devices in long-term knowledge retention [28, 30]. Contrary to this, one recent randomised clinical trial assessing the impact of audience response devices on medical student learning did find that the devices, along with three embedded questions in a 30 min lecture, improved students’ knowledge immediately after the session and again two weeks later [15]. They speculated that this effect was due to forced information retrieval by the students brought on by the learning process [15]. However, most students in this study believed that the “clickers” enhanced their understanding of the topic being covered, but 38% were ambivalent (neither agreed nor disagreed) and this cannot be ignored. Nayak & Erinjeri [31] found there to be a mutual benefit for both the learner and the presenter in teaching sessions involving medical students. Here, and in peer teaching sessions, students indicated that the “clickers” allowed them to gauge the understanding of the audience while in non peer-led interactive sessions the students indicated that the “clickers” gave them more confidence to verbally answer questions in subsequent lectures [31]. Student feedback in our institution indicates that they enjoy MDTs and the “clickers” are a benefit to their own learning. This is consistent with other studies where student attitudes to “clickers” have been evaluated [32, 33].

## Conclusions

From the students’ perspective, the sessions when “clickers” were used were more enjoyable and engaging and the majority perceived the devices to have a positive impact on their understanding of the topic being covered. However, statistically we could not find evidence of a positive impact of the “clickers” on the retention of knowledge or understanding in this study, in this cohort of medical students, after the clinical microbiology focussed MDT. However, “clickers” are a useful tool to promote engagement of undergraduate medical students in this learning environment as they can improve the learning experience for all involved. Such an approach, or others involving newer technologies that utilise applications on smart portable devices for the same purpose, should be considered for large group teaching sessions.

## Appendix

These particular MCQs related to the bacterial meningitis MDT, but the MCQs associated with all other MDTs and online quizzes were structured in the same style.

1. **Which one of the signs is most likely to help differentiate between meningitis and encephalitis?**

A. Cranial nerve palsy
B. Confusion
C. Pharyngitis
D. Photophobia
E. Papilloedema

ANSWER: B

2. **Which one of the following is an indication for a CT brain in a patient presenting with meningitis?**

A. Altered mental status
B. Photophobia
C. Headache
D. Single seizure in childhood
E. Abdominal pain

ANSWER: A

3. **Which one of the following is the commonest cause of meningitis in Ireland?**

A. *Eschericia coli*
B. *Listeria monocytogenes*
C. *Neisseria meningitidis*
D. *Streptococcus pneumoniae*
E. *Staphylococcus aureus*

ANSWER: D

4. **Which one of the following is the best choice for empiric treatment of suspected pneumococcal meningitis?**

A. Benzylpenicillin
B. Cefotaxine plus vancomycin
C. Amoxicillin plus gentamicin
D. Co-amoxiclav
E. Piperacillin-tazobactam

ANSWER: B

5. **Which one of the following is a common complication of bacterial meningitis?**

A. Blindness
B. Cerebral abscess
C. Deafness
D. Ventriculitis
E. Paralysis

ANSWER: C.

## Abbreviations

BSI: Bloodstream infection
CAP: Community-acquired pneumonia
IC: Intermediate Cycle
IE: Infective endocarditis
MCQ: Multiple choice question
MDT: Multidisciplinary teaching
RCSI: The Royal College of Surgeons in Ireland
TB: Tuberculosis
